# Supplementary material for: Interleukin-22 regulates neutrophil recruitment in ulcerative colitis and is associated with resistance to ustekinumab therapy
Source: Nat Commun. 2022 Oct 3;13:5820. doi: 10.1038/s41467-022-33331-8 (PMC9530232; doi:10.1038/s41467-022-33331-8)
Supplement: Supplementary file 1 — Supplementary Information [file 41467_2022_33331_MOESM1_ESM.pdf]

Supplementary Figure 1

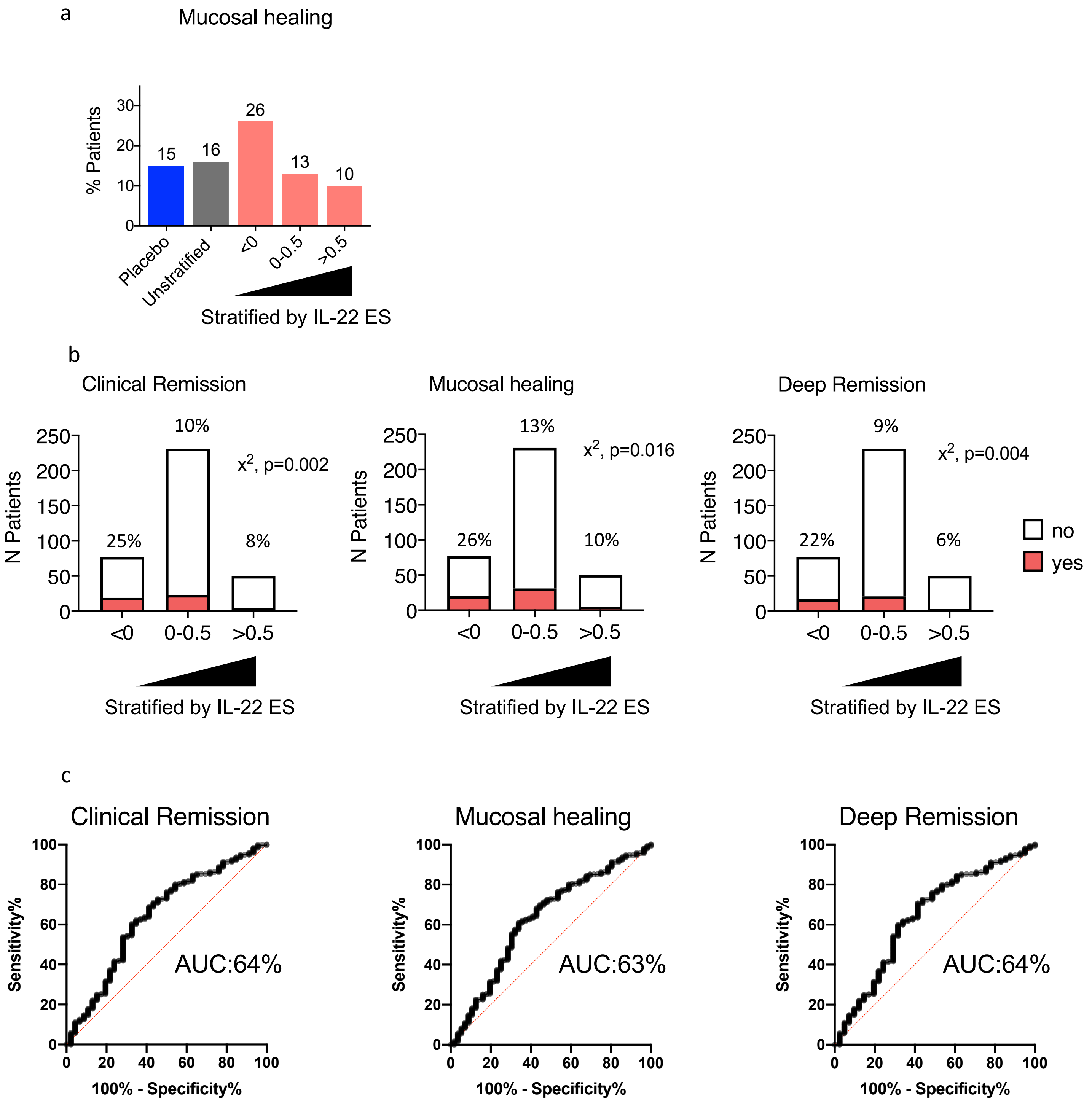

Supplementary Figure 1. Expression of IL-22 transcriptional modules in colonic biopsies associates with response to ustekinumab in ulcerative colitis. (a) Mucosal healing defined as histologic improvement (neutrophil infiltration in <5% of crypts, no crypt destruction, and no erosions, ulcerations, or granulation tissue) and endoscopic improvement (total Mayo score of ≤2 and no subscore >1) at week 8 in UC patients enrolled in the UNIFI clinical trial program stratified according to IL22 enrichment score in baseline biopsies sampled immediately prior to initiation of ustekinumab or placebo. (b) Outcome rates as number of patients and frequency per IL-22 enrichment score (ES) stratum in ustekinumab treated patients (UNIFI, n=358, contingency table- x^2 analysis, two tailed test). (c) Receiver operator characteristic curve analysis for the IL22 enrichment score to predict outcomes at week 8 in ustekinumab treated patients participating in UNIFI (n=358), AUC: area under the curve. Source data for a, b and c are provided as a Source Data file.

Supplementary Figure 2

a

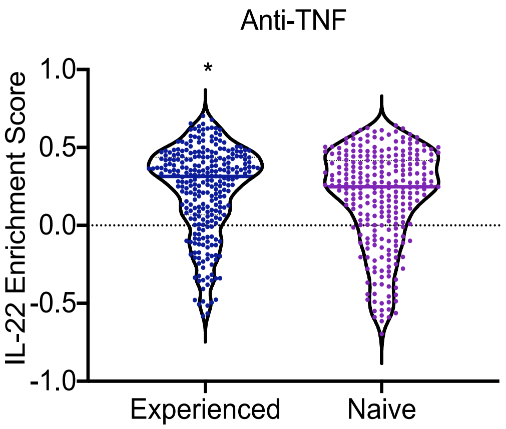

b

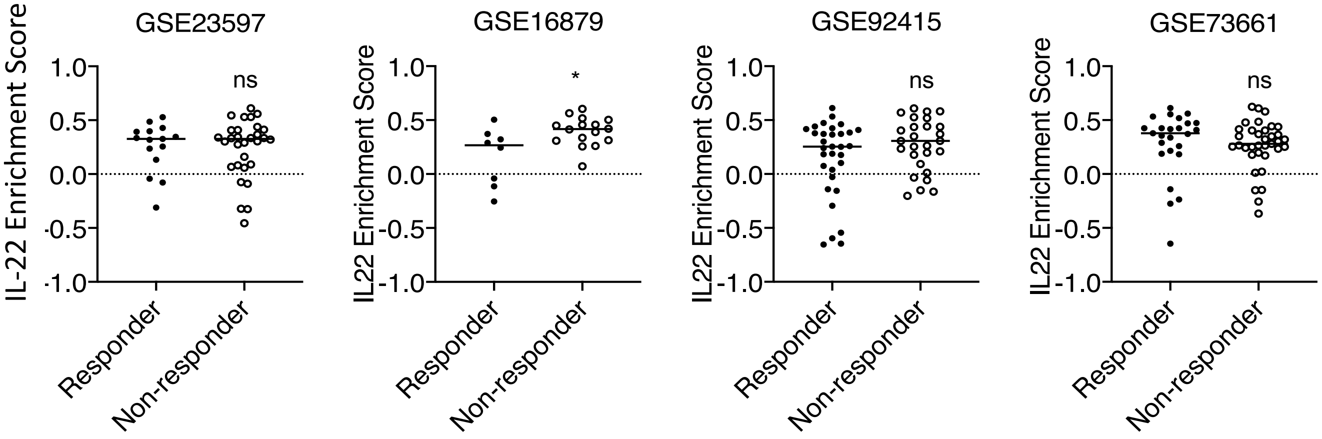

GEO	GSE23597	GSE16879	GSE92415	GSE73661
n	45	24	59	60
drug	infliximab	infliximab	golimumab	vedolizumab
time point	wk8	wk8	wk6	wk4-6
endpoint	clinical response	mucosal healing	clinical response	mucosal healing

Supplementary Figure 2. IL-22 regulated transcriptional program and response to other biologics in UC. (a) IL-22 enrichment score in anti-TNF naive and experienced (failure) patients participating in UNIFI (n= 550, Mann Whitney, one-tailed, *p=0.032, line representing median) (b) IL-22 enrichment score in biopsies of patients who were treated with an anti-TNF (GSE23597/GSE16879-infliximab, GSE92415-golimumab and GSE73661-vedolizumab, wk: week, Mann Whitney test, two-tailed, *p<0.05). Source data for a and b are provided as a Source Data file.

Supplementary Figure 3

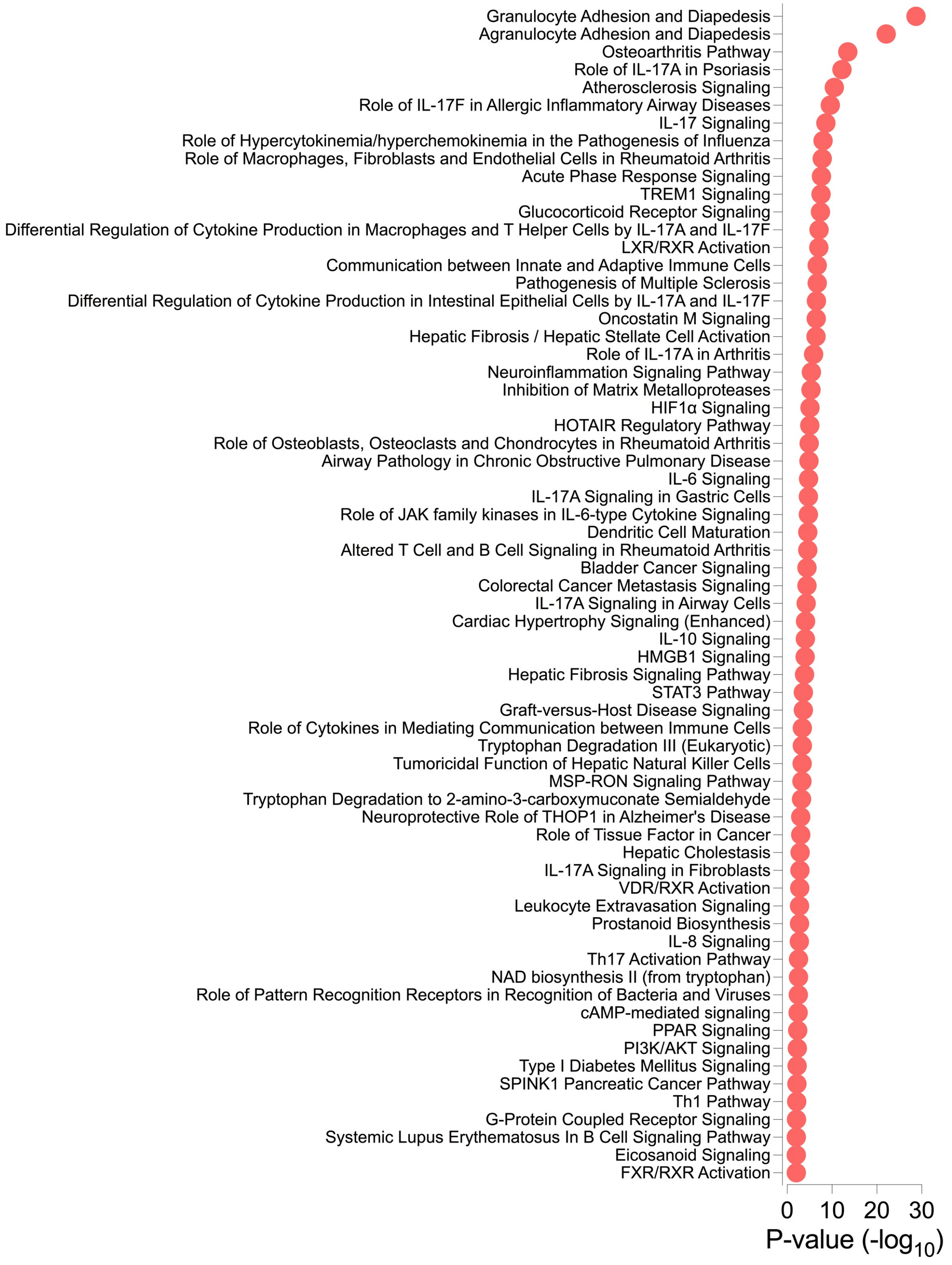

Supplementary Figure 3. Top, most significantly associated canonical pathways in patients with high IL-22 enrichment scores (ES) defined as IL-22 ES>0.25 (right tailed Fisher exact test), Ingenuity Pathway Analysis, IPA). Source data are provided as a Source Data file.

Supplementary Figure 4

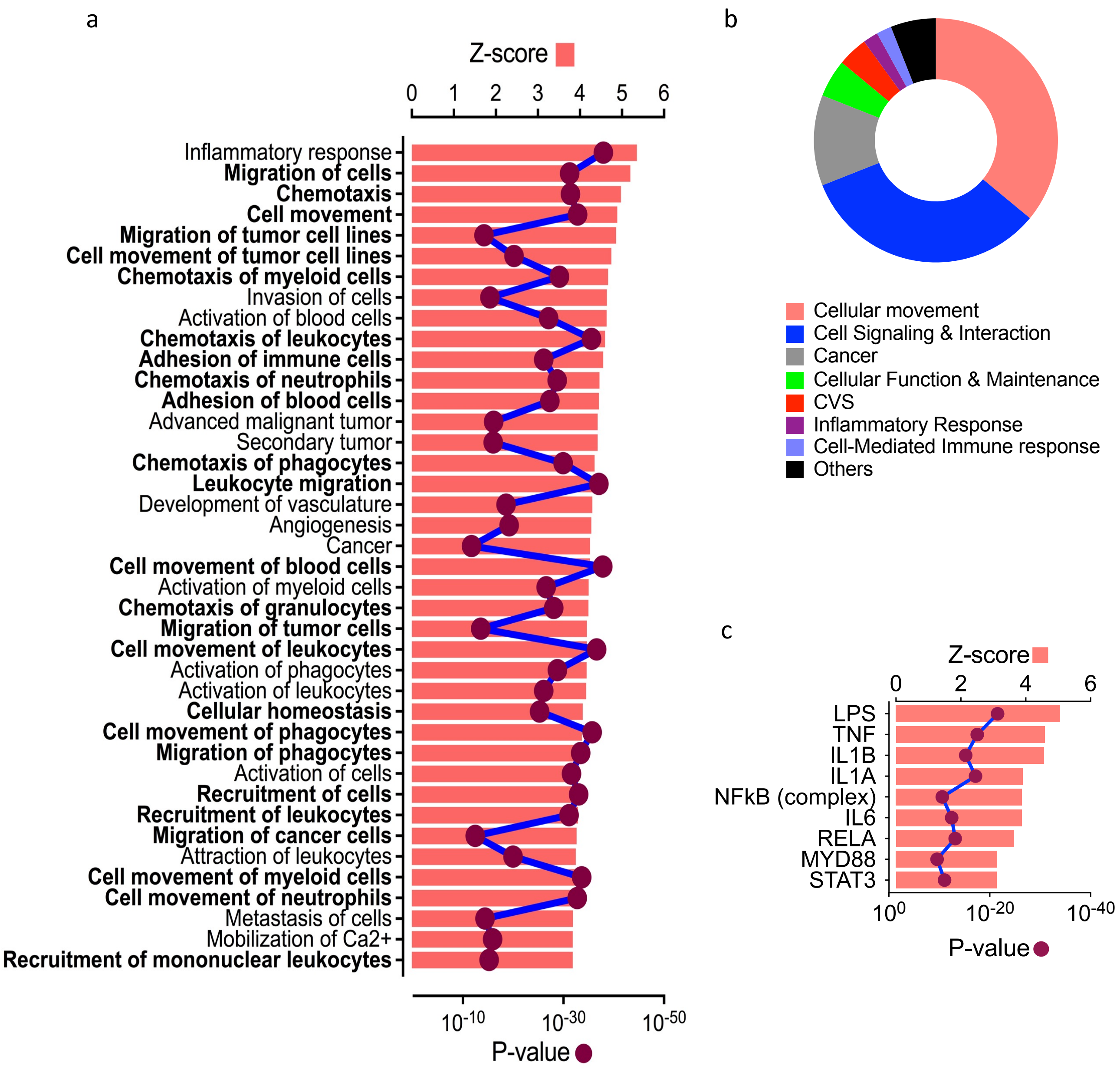

Supplementary Figure 4: Bioinformatic analysis predicts causal effects and biological processes activated in patients with high IL-22 enrichment scores. The IPA (ingenuity) Downstream Effects Analysis algorithm was used to define biological pathways activated in patients from the UNIFI cohort with high (IL-22 ES>0.25) and low IL-22 enrichment scores (IL-22 ES<0.25). (a) Top 40 pathways are identified and pathways involving cell trafficking are highlighted in bold (right tailed Fisher exact test). (b) Proportion of annotations with different high level categories in Molecular and Cellular Functions. (c) IPA upstream regulator analysis identified LPS, TNF and IL-1β as strongly predicted activators of the transcriptional program observed in patients with high IL-22 enrichment scores (right tailed Fisher exact test). Regulator effects analysis was performed on the top 3 predicted upstream activators (also see Figure 2C). Source data are provided as a Source Data file.

Supplementary Figure 5

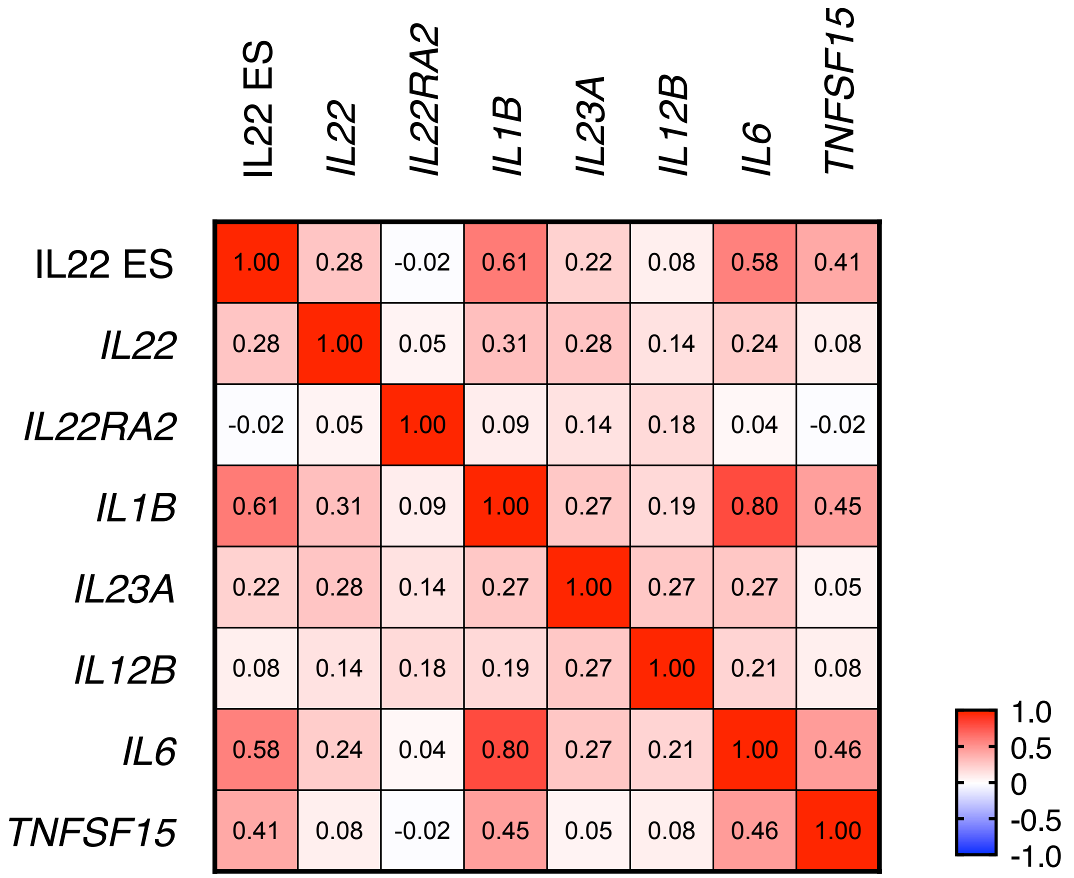

Supplementary Figure 5. UC stratification by the IL-22 regulated transcriptional program is associated with key upstream regulators of IL-22 expression. Correlation matrix (non-parametric Spearman r, two-tailed) of the key upstream regulators for *IL22* and the IL22 enrichment score (ES) including the expression of *IL22RA2*, coding the IL-22 binding protein (IL22BP), (UNIFI, UC, n=550). Source data are provided as a Source Data file.

Supplementary Figure 6

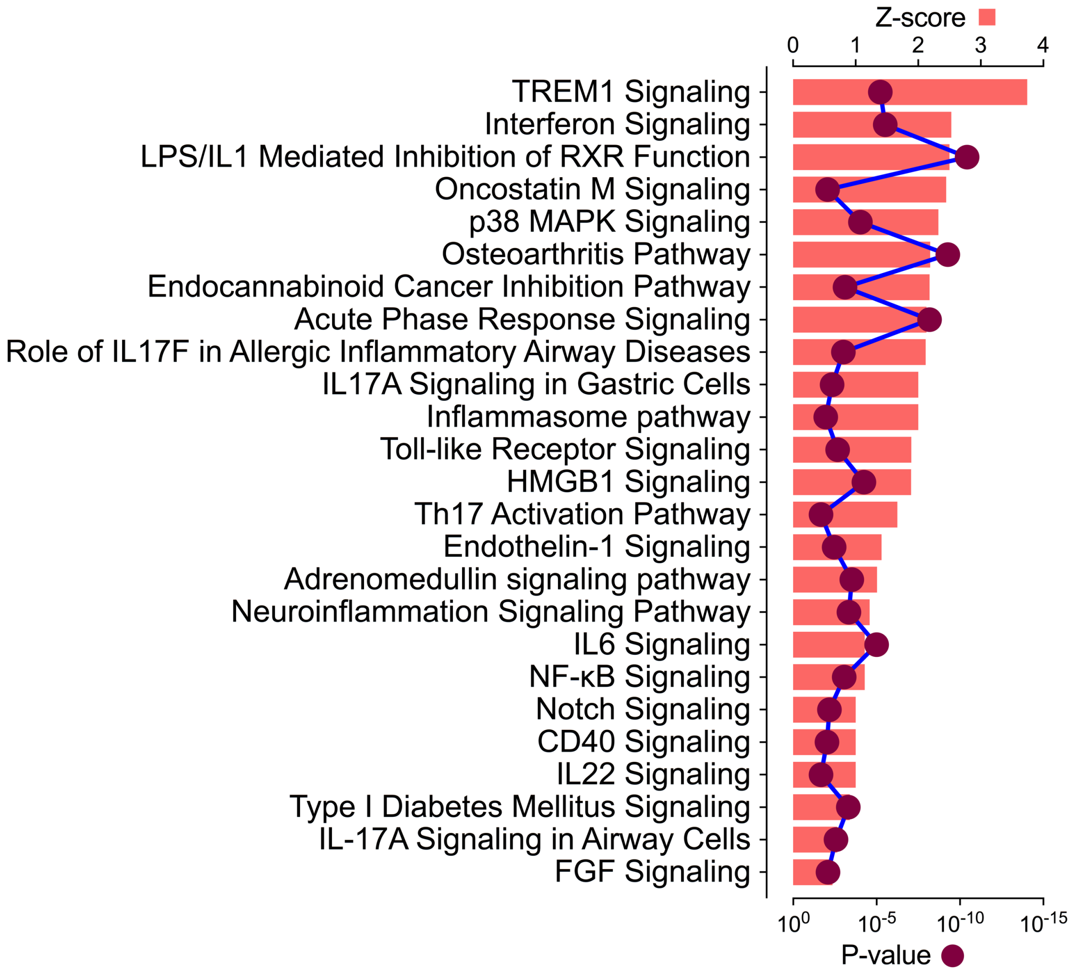

Supplementary Figure 6: Canonical pathway analysis of the IL-22 transcriptional program. IPA canonical pathway analysis of the IL-22 regulated transcriptional programme, top 25 results shown (right tailed Fisher exact test).

Supplementary Figure 7

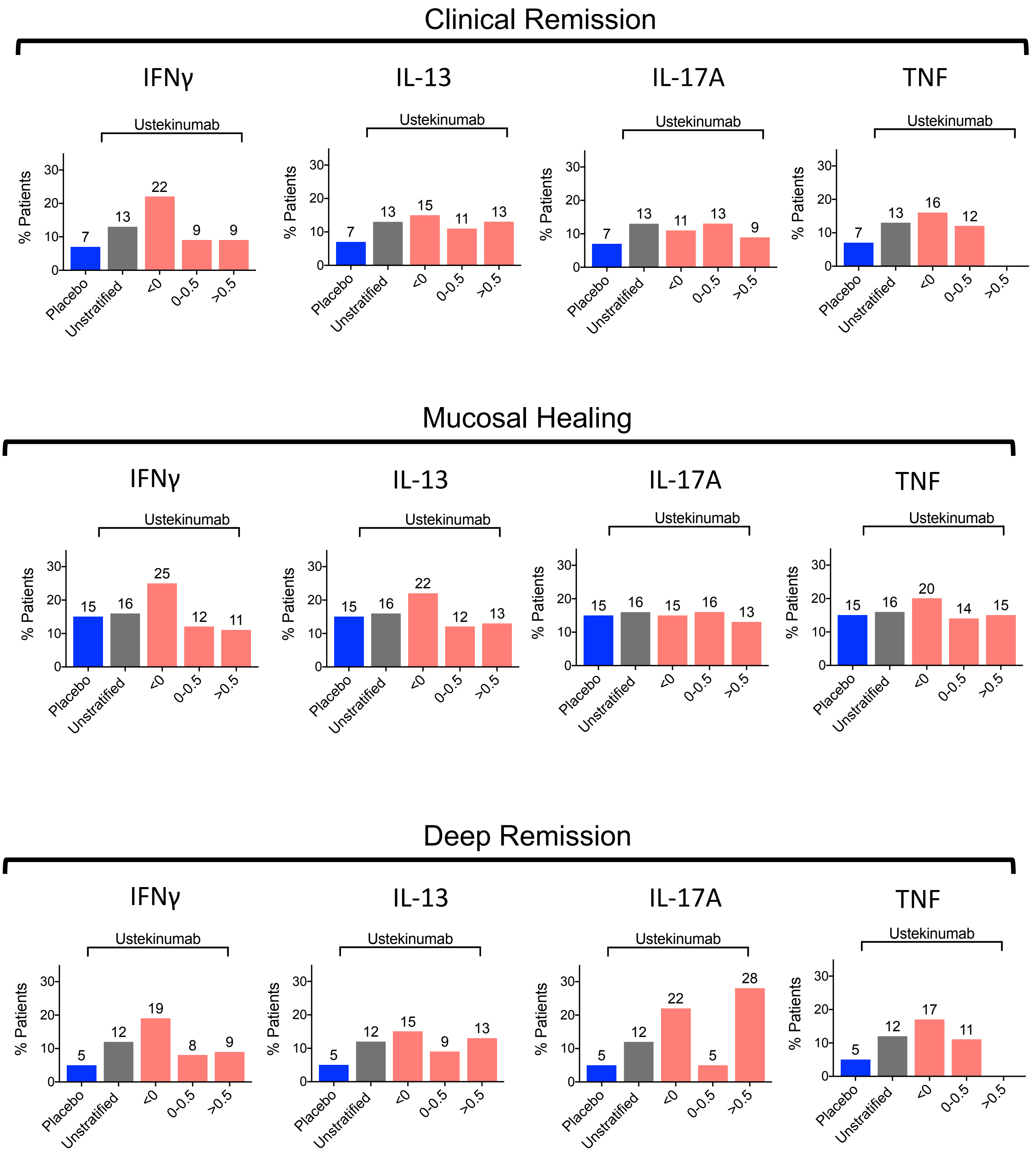

Supplementary Figure 7. Expression of cytokine regulated transcriptional modules in colonic biopsies and association to response to ustekinumab in ulcerative colitis. (A) Clinical remission (defined as a total Mayo score of ≤2 and no subscore >1), mucosal healing defined as histologic improvement (neutrophil infiltration in <5% of crypts, no crypt destruction, and no erosions, ulcerations, or granulation tissue) and endoscopic improvement (total Mayo score of ≤2 and no subscore >1) and deep remission (which required both clinical remission and mucosal healing) at week 8 in UC patients enrolled in the UNIFI clinical trial program stratified according to cytokine regulated transcriptional programme enrichment score in baseline biopsies sampled immediately prior to initiation of ustekinumab (n=358) or placebo(n=184). Source data are provided as a Source Data file.

Supplementary Figure 8

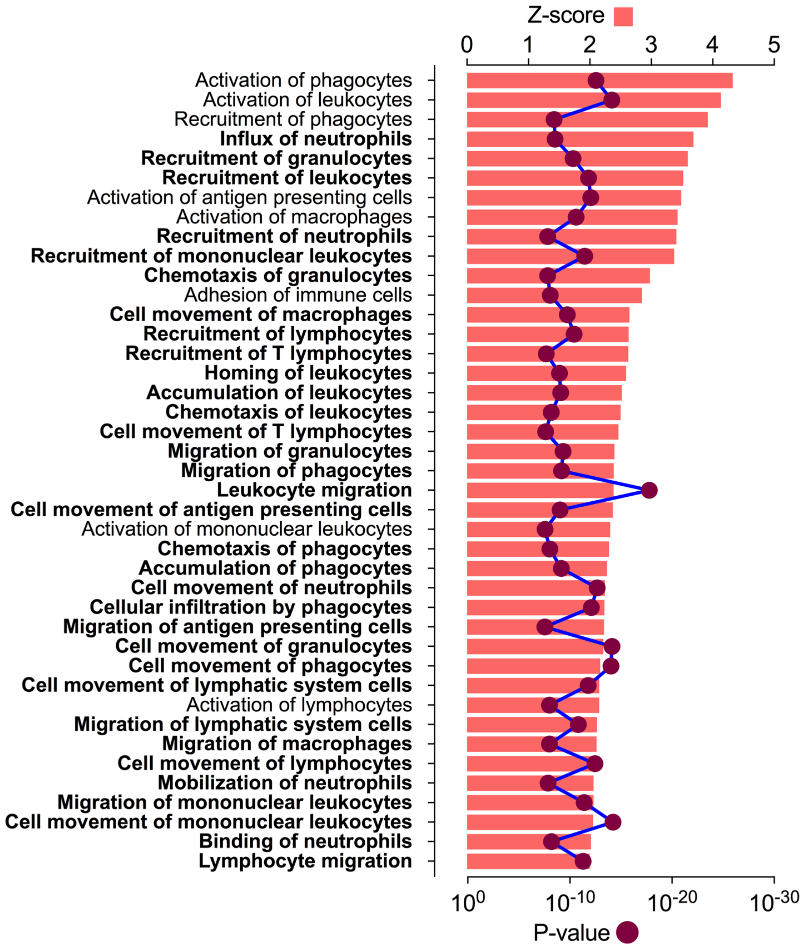

Supplementary Figure 8: Pathway analysis highlights neutrophil chemotaxis as key pathway regulated by IL-22. Diseases & Functions analysis of the IL-22 regulated transcriptional programme with IPA (Ingenuity), pathways comprising the immune cell trafficking pathway are shown (right tailed Fisher exact test).

Supplementary Figure 9

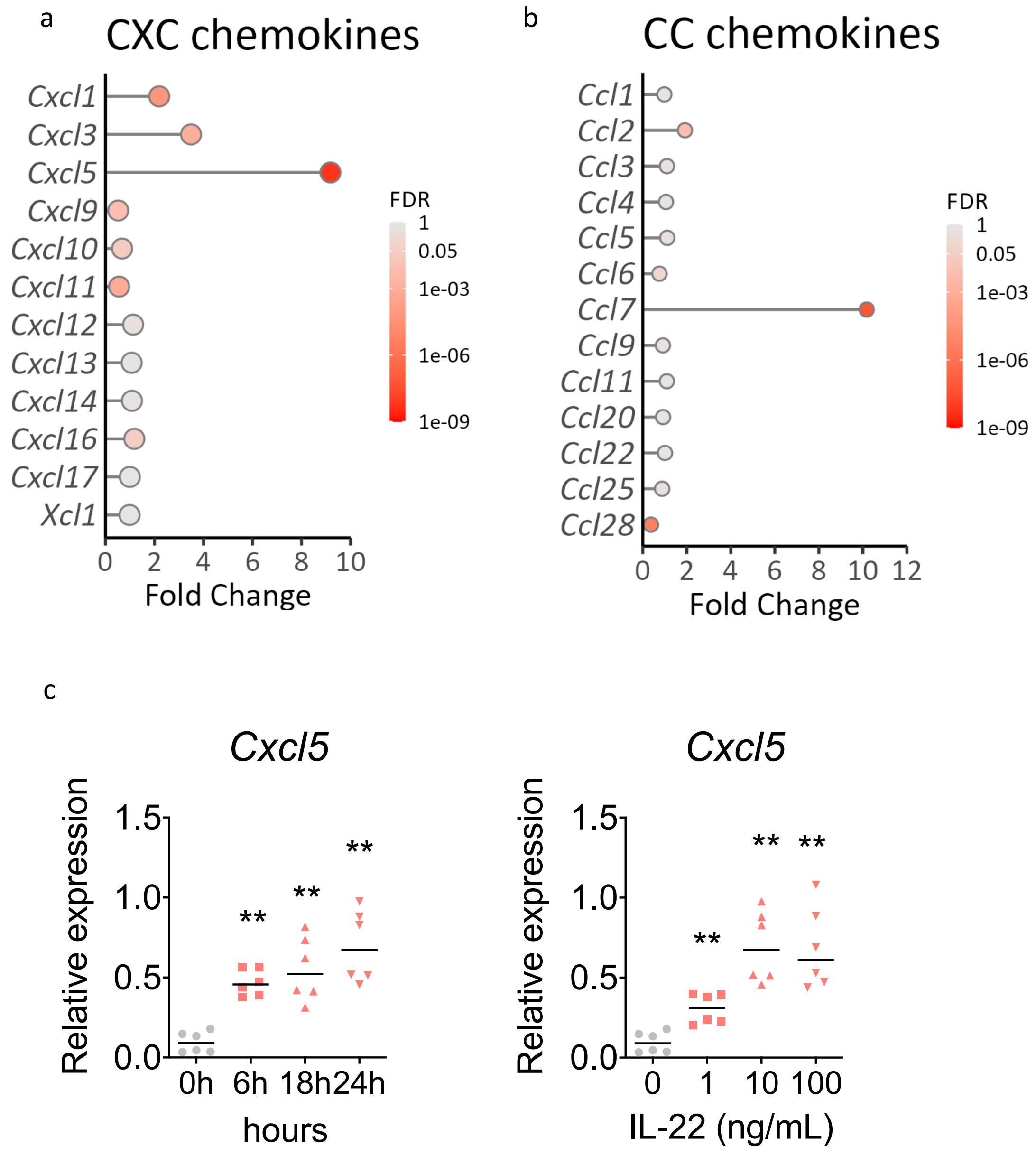

Supplementary Figure 9: Selective regulation of neutrophil attracting chemokines in mouse colonoids by IL-22. Relative expression of CXC (a) and CC (b) chemokine transcripts in mouse colonic organoids treated with IL-22 for 24hrs (n=5, biological replicates). (c)Validation of the RNAseq experiment using a commercially available primer for the neutrophil attracting chemokine *Cxcl5* (IL22 dose for time course: 10ng/mL). A positive time/dose association with IL-22 treatment was observed (n=6, biological replicates, Mann-Whitney, two tailed test, comparing each time point or concentration with baseline, **p=0.002, line denotes: median). Source data for a, b and c are provided as a Source Data file.

Supplementary Figure 10

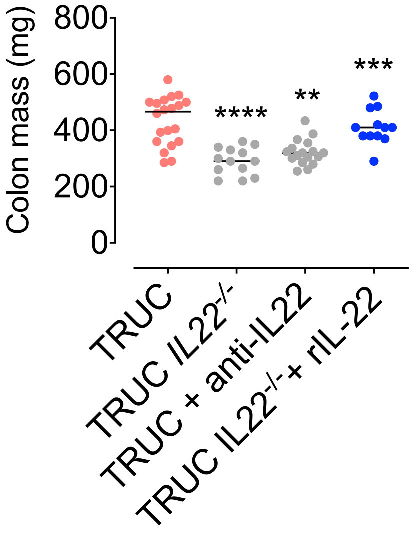

Supplementary Figure 10: Pathogenic role of the IL22 mediated neutrophil chemotaxis axis in TRUC. Loss or blockade of IL22 alleviate TRUC disease while supplementation with recombinant IL22 in the IL22 knock out TRUC model recapitulates disease (n=36, biological replicates, Kruskal Wallis with Dunn's multiple comparison test, TRUC *IL22-/- vs* TRUC, TRUC+anti_IL22 *vs.* TRUC , ****p<0.0001, **p<0.01 and Mann Whitney, two tailed test for TRUC IL22-/-+rIL-22 *vs.* TRUC+anti-IL22, ***p<0.001, line denotes median). Neutralizing anti-IL-22 mAb (clone IL22-01, 200µg per mouse) were administered intraperitoneally (ip) every 3 to 4 days. Recombinant IL22 (rIL22, 100µg per mouse) were administered ip at days 0, 4, 8 and 12. Source data are provided as a Source Data file.

Supplementary Figure 11

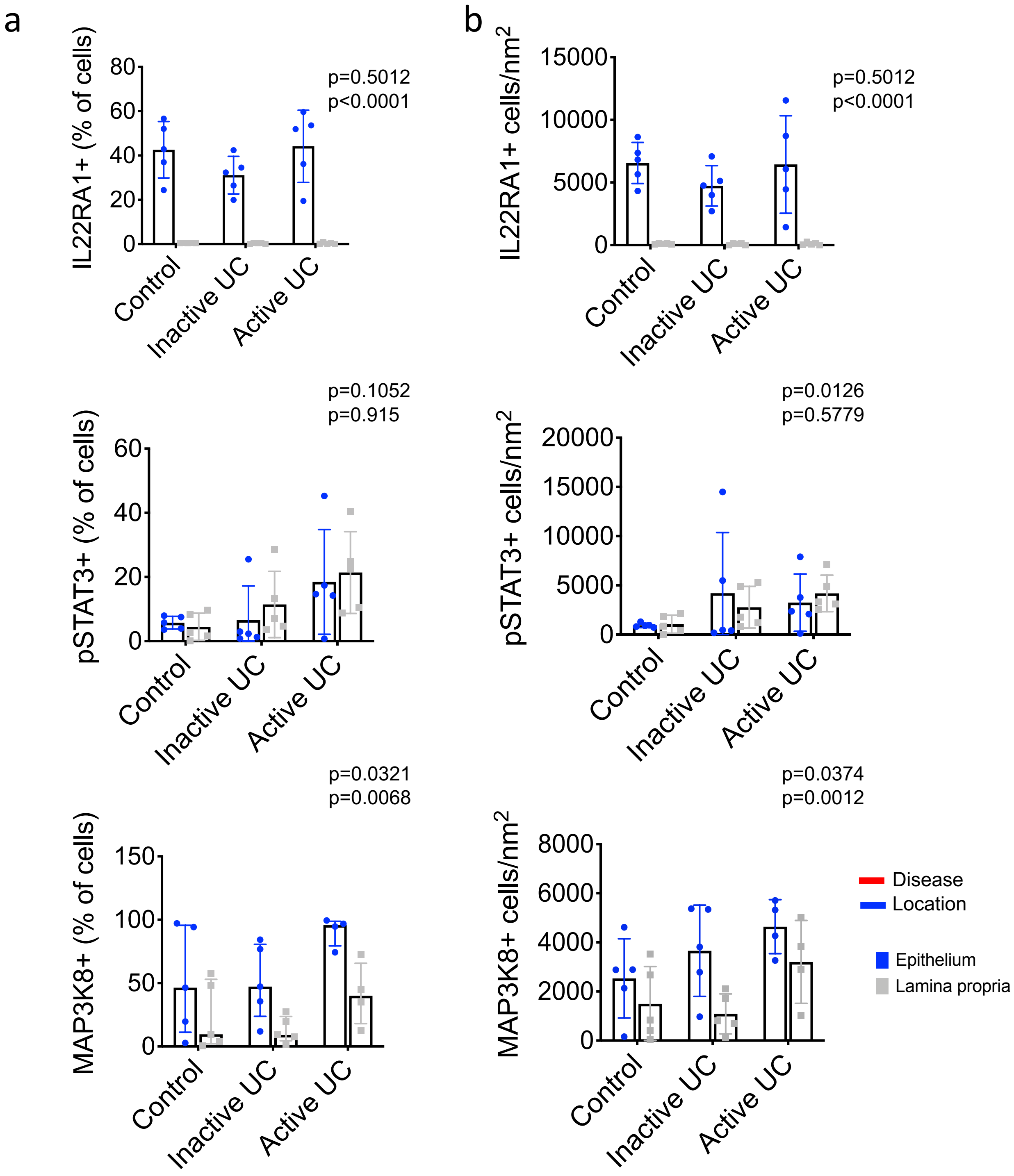

Supplementary Figure 11: Immunostaining of IL22RA1, pSTAT3 and MAP3K8 in human colonic biopsies (a)reported as cells/nm^2, (b) reported as cell frequency, n=15 (five individuals per group), two-way ANOVA, mean with standard deviation, p values for comparison as per disease or location are provided. Source data for a and b are provided as a Source Data file.

Supplementary Figure 12

Representative gating strategy for LP Neutrophils

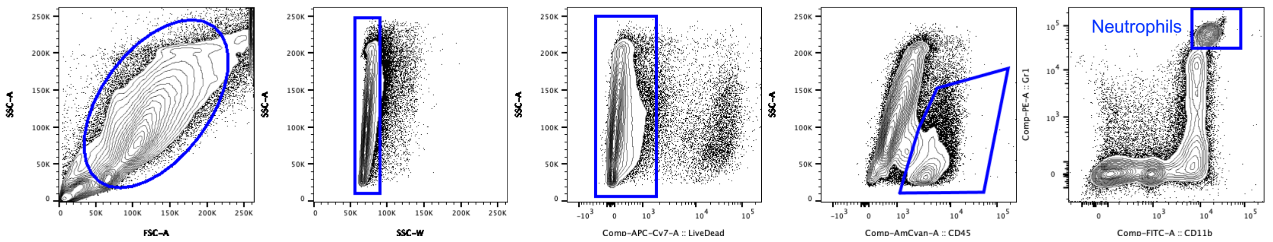

Supplementary Figure 12: Representative gating strategy for lamina propria neutrophils.

Supplementary Figure 13

Representative gating strategy for NCR$^-$ ILC3 FACS

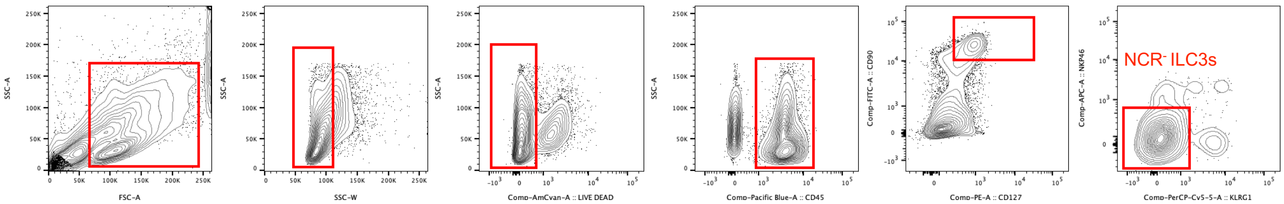

Supplementary Figure 13: Representative gating strategy for NCR-ILC3 fluorescence-activated cell sorting.

	UC
n	550
age	42
sex (F:M)	35% : 65%
Mayo score	9 (8,12)
Disease duration (years)	6 (3, 12)
Previous antiTNF	50%
CRP	4.6 (1.4, 12)
Faecal Calprotectin (ug/g)	1310 (560, 2594)
Faecal Lactoferrin (ug/g)	184 (60, 455)

Supplementary Table 1 : UNIFI cohort- demographic data [median followed by range (min, max)].

IFNGtop50	IL17Atop50	IL22top50	TNFatop50	IL13top50
IDO1	SAA1	DMBT1	C3	CCL26
CXCL10	SNPH	SERPINA3	CYP2A6	ALOX15
CXCL9	SERPINB7	REG1A	SERPINA3	TREML2
GBP5	FABP6	REG1B	SERPINB7	CDH26
NOS2	LCN2	HTRA1	TMEM119	LCT
GBP4	SAA2	NOS2	DRGX	ITGA2B
SERPING1	IL1A	SOCS3	CLEC2D	MMP1
HLA-DPA1	CCL20	CFI	NPTX1	FLT1
MS4A18	IL17C	DSG3	CYP4Z1	CAPN13
WARS	CXCL5	PLA2G2A	CCL2	SH2D1B
CD274	PI3	TNIP3	KLHDC7B	LGR6
PLA2G2A	PDZK1IP1	TIFA	CYP2A7	WNT5B
CIITA	VSIG1	HTR3A	TNFRSF9	FHOD3
OAS2	CA4	LYPD1	SPIB	SLC26A4
HAPLN3	PPBP	IFITM3	NCCRP1	SLIT2
IL18BP	NOS2	TGM2	CYP4X1	SOCS1
CX3CL1	MIR146A	SLC9B2	LTB	POMC
CD7	LGALS2	IFITM1	UNC13A	FSTL4
IFI44L	DUOXA2	PDZK1IP1	CX3CL1	WFDC21P
HLA-DPB1	MTNR1A	GRIN3A	SERPINA5	AIF1
HLA-DQA1	IL13RA2	SERPINA1	CYP2B7P	SNPH
CXCL11	IL1RL1	ALPPL2	IL36B	ANKRD33B
HLA-DRA	CXCL6	STMN3	ALOX15B	C1QTNF1
GBP1P1	CSF2	WNT5A	GBP4	CCL24
CPA2	FSTL1	SLC5A8	CSF2	SORCS2
ZBP1	DNM3	OLFM4	TTN	MUC6
HLA-DMB	MMP10	HEG1	NPR1	DNAH9
BST2	VNN2	FSTL1	SLC30A2	NPR1
TRPV6	PRRX2	FLNC	PI3	RGS2
HLA-DOA	SEMG1	ENKUR	PRRX2	C1QTNF1-AS1
LGALS17A	SH2D1B	C2CD4A	UBD	ADAM19
IFIT3	VNN1	CARD14	MYH7	TMOD2
GBP1	ANXA6	GPR37	MMP10	LINC01913
CHRD	ZC3H12C	MMP10	DACT2	CYP2B6
SECTM1	CXCL1	IL1R1	SPX	CCL15
ISG15	CCL28	CITED4	ICAM1	ATP12A
MDGA1	APOA1	PRUNE2	TNF	TRPV6
CD74	CXCL8	ECEL1P2	LAYN	LINC00330
EPSTI1	DUOX2	GBP4	SCNN1B	GJB6
RARRES3	LRRTM1	KCNN2	SLC5A2	LBH
CMPK2	DEFB1	CASP5	ARHGAP31	DNAH12
RSAD2	MMP7	TRPV6	KCNE1	HSD3B1
CCL22	ANXA1	KRT6A	EGOT	LINC02577
UBE2L6	PKIG	NAV3	ESYT3	NLRP3
MXRA5	CPNE5	TMEM173	NEB	SNORD104
HLA-DQB1	C4BPA	OSMR	SV2B	CD7
MUC16	ADTRP	STEAP4	CCL22	SNCAIP
MMP25	ZC3H12A	IL13RA2	CHST2	LOC101927531
IFITM1	RND1	PLEKHF1	IL23A	OVOS2
LYPD5	MT1G	LYPD5	PLAU	CFI

Supplementary Table 2: Cytokine gene signatures defined as the top 50 upregulated transcripts by each cytokine in human colonoids

**Chemicals, Peptides, Recombinant Proteins and Monoclonal Antibodies**		
Advanced DMEM/F-12	Gibco	12634010
GlutaMAX	Gibco	11574466
HEPES	Gibco	15630049
Penicillin/Streptomycin	Sigma-Aldrich	P4333
Wnt3a conditioned medium	In-house production	-
R-spondin 1 conditioned medium	In-house production	-
Recombinant murine Noggin	Peprotech	250-38-100
B27 Supplement	Gibco	17504044
N2 Supplement	Gibco	17502048
N-Acetylcysteine	Sigma-Aldrich	A9165
EGF Recombinant Mouse Protein	Gibco	PMG8041
Nicotinamide	Sigma-Aldrich	N0636
Gastrin	Sigma-Aldrich	G9145
A 83-01	Bio-Techne	2939
SB-202190	Sigma-Aldrich	S7067
Y-27632	Sigma-Aldrich	Y0503
CHIR99021	Sigma-Aldrich	SML1046
EDTA	Invitrogen	15575-038
Matrigel	Corning	356231
Cell Recovery Solution	Corning	354253
RNase-Free DNase Set	Qiagen	79254
RPMI	Gibco	11875-093
Collagenase D	Roche	11088882001
Dispase II	Roche	4942078001
DNAse	ROCHE	10104159001
Percoll	GE Healthcare	17-0891-01
Recombinant human IL-17A	Bio-Techne	7955-IL
Recombinant human TNF	Bio-Techne	210-TA
Recombinant human IFNγ	Bio-Techne	285-IF
Recombinant human IL-13	Bio-Techne	213-ILB
Recombinant mouse IL-2	Biolegend	589102
Recombinant mouse IL-7	Biolegend	577802
Recombinant mouse IL-23	Biolegend	589002
Recombinant IL-1β	Biolegend	401-ML-005
anti-IL22	Pfizer	Clone IL22-01
Recombinant IL-22	Pfizer	(not commercially available)
Anti-CXC2	R&D Systems	Clone 242216
Anti-IL22RA	Novus Biologicals	NBP2-38496
pSTAT3	RabMAb	EP2147Y (ab76315)
MAP3K8	Thermo Fisher	PA5-21650

**Commercial assays and primers**		
RNeasy Mini Kit	Qiagen	74104
Qubit RNA BR assay kit	Thermo Fisher Scientific	Q10210
Bioanalyzer RNA 6000 Nano Kit	Agilent	5067-1511
NEBNext® UltraTM RNA Library Prep Kit for Illumina®	New England Biolabs	E7530L
HG U133 PM array	Affymetrix	
CXCL1 Elisa kit	R&D Systems	DY453
CXCL5 Elisa kit	R&D Systems	DY443
Cxcl1 primer	IDT Technologies	Designed to order
Cxcl5 primer	IDT Technologies	Designed to order

**Flow cytometry reagents**					
Antigen	Fluorochrome	Clone	Source	Cat No	Dilution
CD4	BV786	RM4-5	BD Biosciences	563727	1:200
CD4	FITC	GK1.5	BD Biosciences	553729	1:200
CD11b	FITC	M1/70	BD Biosciences	568688	1:200
CD25	APC	PC61.5	eBioscience	17-0251-82	1:200
CD44	PE	IM7	eBioscience	12-0441-82	1:200
CD45	Pac orange	30-F11	Invitrogen	MCD4530	1:200
CD45	V500	30-F11	BD Biosciences	561487	1:200
CD62L	Pac Blue	MEL-14	Biolegend	104424	1:200
CD127	APC	A7R34	eBioscience	17-1271-82	1:200
CD127	BUV737	SB/199	BD Biosciences	612841	1:200
Gr-1	PE	RB6-8C5	eBioscience	12-5931-82	1:200
Gr-1	APC-Cy7	RB6-8C5	eBioscience	A15424	1:200
KLRG1	PerCP-eFlour710	2F1	eBioscience	46-5893-82	1:100
NKp46	PE-Cy7	29A1.4	eBioscience	25-3351-82	1:200

Supplementary Table 3: Reagent list
