# Supplementary material for: Interleukin-22 regulates neutrophil recruitment in ulcerative colitis and is associated with resistance to ustekinumab therapy
Source: Nat Commun. 2022 Oct 3;13:5820. doi: 10.1038/s41467-022-33331-8 (PMC9530232; doi:10.1038/s41467-022-33331-8)
Supplement: Supplementary file 3 — Reporting Summary [file 41467_2022_33331_MOESM3_ESM.pdf]

Corresponding author(s): NCOMMS-20-41411C

Last updated by author(s): 25.07.2022

Reporting Summary

Nature Portfolio wishes to improve the reproducibility of the work that we publish. This form provides structure for consistency and transparency in reporting. For further information on Nature Portfolio policies, see our Editorial Policies and the Editorial Policy Checklist.

Statistics

For all statistical analyses, confirm that the following items are present in the figure legend, table legend, main text, or Methods section.

n/a	Confirmed	
☐	☒	The exact sample size (*n*) for each experimental group/condition, given as a discrete number and unit of measurement
☐	☒	A statement on whether measurements were taken from distinct samples or whether the same sample was measured repeatedly
☐	☒	The statistical test(s) used AND whether they are one- or two-sided<br>*Only common tests should be described solely by name; describe more complex techniques in the Methods section.*
☐	☒	A description of all covariates tested
☐	☒	A description of any assumptions or corrections, such as tests of normality and adjustment for multiple comparisons
☐	☒	A full description of the statistical parameters including central tendency (e.g. means) or other basic estimates (e.g. regression coefficient) AND variation (e.g. standard deviation) or associated estimates of uncertainty (e.g. confidence intervals)
☐	☒	For null hypothesis testing, the test statistic (e.g. *F*, *t*, *r*) with confidence intervals, effect sizes, degrees of freedom and *P* value noted<br>*Give P values as exact values whenever suitable.*
☐	☒	For Bayesian analysis, information on the choice of priors and Markov chain Monte Carlo settings
☒	☐	For hierarchical and complex designs, identification of the appropriate level for tests and full reporting of outcomes
☐	☒	Estimates of effect sizes (e.g. Cohen's *d*, Pearson's *r*), indicating how they were calculated

Our web collection on statistics for biologists contains articles on many of the points above.

Software and code

Policy information about availability of computer code

Data collection	No software was used for data collection
Data analysis	DEG analysis for the colonoid experiment was based on a varying intercepts hierarchical modelling approach implemented in R and Stan. Pathway analysis was perfomed with Ingenuity Pathway Analysis (IPA, Qiagen). Protein protein interaction analysis was undertaken in Cytoscape utilizing the STRING database. Gene set enrichment was performed using the GSVA and clusterprofiler packages in R. GraphPad Prism 8 was used for data analysis and graph generation.

For manuscripts utilizing custom algorithms or software that are central to the research but not yet described in published literature, software must be made available to editors and reviewers. We strongly encourage code deposition in a community repository (e.g. GitHub). See the Nature Portfolio guidelines for submitting code & software for further information.

Data

Policy information about availability of data

All manuscripts must include a data availability statement. This statement should provide the following information, where applicable:

- Accession codes, unique identifiers, or web links for publicly available datasets
- A description of any restrictions on data availability
- For clinical datasets or third party data, please ensure that the statement adheres to our policy

The human organoid data generated in this study have been deposited in the Gene Expression Omnibus database under accession codes GSE190705 [https://www.ncbi.nlm.nih.gov/geo/query/acc.cgi?acc=GSE190705] and GSE190634 [https://www.ncbi.nlm.nih.gov/geo/query/acc.cgi?acc=GSE190634]. The mouse organoid data generated in this study have been deposited in the Gene Expression Omnibus database under accession code ZZ [add hyperlink here]. The mouse colitis models data generated in this study have been deposited in the Gene Expression Omnibus database under accession code ZZ [add hyperlink here].

The datasets generated during and/or analysed during the current study are available in the GEO repository:
https://www.ncbi.nlm.nih.gov/geo/query/acc.cgi?acc=GSE190705
https://www.ncbi.nlm.nih.gov/geo/query/acc.cgi?acc=GSE190634
https://www.ncbi.nlm.nih.gov/geo/query/acc.cgi?acc=GSE59071
https://www.ncbi.nlm.nih.gov/geo/query/acc.cgi?acc=GSE23597
https://www.ncbi.nlm.nih.gov/geo/query/acc.cgi?acc=GSE16879
https://www.ncbi.nlm.nih.gov/geo/query/acc.cgi?acc=GSE92415
https://www.ncbi.nlm.nih.gov/geo/query/acc.cgi?acc=GSE73661

Field-specific reporting

Please select the one below that is the best fit for your research. If you are not sure, read the appropriate sections before making your selection.

- [x] Life sciences
- [ ] Behavioural & social sciences
- [ ] Ecological, evolutionary & environmental sciences

For a reference copy of the document with all sections, see nature.com/documents/nr-reporting-summary-flat.pdf

Life sciences study design

All studies must disclose on these points even when the disclosure is negative.

Sample size	No formal sample size calculation was performed prior to the study. Summary statistics with confidence intervals provide effect sizes and estimate of confidence for interpretation
Data exclusions	No data were excluded
Replication	Our analysis of the ustekinumab treated clinical trial cohort was performed separately by KL, FFY and PP, NP confirming same results. This is currently the largest cohort of its kind with wide representation of patients based on their demographics, as previously reported (NEJM 2019). Where necessary replicate experiments were performed to validate reproducibility of findings.
Randomization	Patients were randomized to placebo or ustekinumab treatment as per protocol for the phase 3 registration clinical trial (NEJM 2019). Selection of mice for animal experiments was random. Patients who provided samples for organoid generation and trasncriptomic profiling of cytokine responses were randomly chosen.
Blinding	Blinding was applied to histology and immunohistochemistry reporting.

Reporting for specific materials, systems and methods

We require information from authors about some types of materials, experimental systems and methods used in many studies. Here, indicate whether each material, system or method listed is relevant to your study. If you are not sure if a list item applies to your research, read the appropriate section before selecting a response.

Materials & experimental systems

n/a	Involved in the study	
[ ]	[x]	Antibodies
[x]	[ ]	Eukaryotic cell lines
[x]	[ ]	Palaeontology and archaeology
[ ]	[x]	Animals and other organisms
[ ]	[x]	Human research participants
[ ]	[x]	Clinical data
[x]	[ ]	Dual use research of concern

Methods

n/a	Involved in the study	
[x]	[ ]	ChIP-seq
[ ]	[x]	Flow cytometry
[x]	[ ]	MRI-based neuroimaging

Antibodies

Antibodies used

Antigen Fluorochrome Clone Source Cat No
CD4 BV786 RM4-5 BD Biosciences 563727
CD4 FITC GK1.5 BD Biosciences 553729
CD11b FITC M1/70 BD Biosciences 568688
CD25 APC PC61.5 eBioscience 17-0251-82
CD44 PE IM7 eBioscience 12-0441-82
CD45 Pac orange 30-F11 Invitrogen MCD4530
CD45 V500 30-F11 BD Biosciences 561487
CD62L Pac Blue MEL-14 Biolegend 104424

CD127 APC A7R34 eBioscience 17-1271-82
CD127 BUV737 SB/199 BD Biosciences 612841
Gr-1 PE RB6-8C5 eBioscience 12-5931-82
Gr-1 APC-Cy7 RB6-8C5 eBioscience A15424
KLRG1 PerCP-eFlour710 2F1 eBioscience 46-5893-82
NKp46 PE-Cy7 29A1.4 eBioscience 25-3351-82

Validation

https://www.bdbiosciences.com/en-tw/products/reagents/flow-cytometry-reagents/research-reagents/single-color-antibodies-ruo/bv786-rat-anti-mouse-cd4.563727
https://www.bdbiosciences.com/ko-kr/products/reagents/flow-cytometry-reagents/research-reagents/single-color-antibodies-ruo/fitc-rat-anti-mouse-cd4.553729
https://www.bdbiosciences.com/en-us/products/reagents/flow-cytometry-reagents/research-reagents/single-color-antibodies-ruo/fitc-rat-anti-cd11b.561688
https://www.thermofisher.com/antibody/product/CD25-Antibody-clone-PC61-5-Monoclonal/17-0251-82
https://www.thermofisher.com/antibody/product/CD44-Antibody-clone-IM7-Monoclonal/12-0441-82
https://www.thermofisher.com/antibody/product/CD45-Antibody-clone-30-F11-Monoclonal/MCD4530
https://www.bdbiosciences.com/en-us/products/reagents/flow-cytometry-reagents/research-reagents/single-color-antibodies-ruo/v500-rat-anti-mouse-cd45.561487
https://www.biolegend.com/it-it/search-results/pacific-blue-anti-mouse-cd62l-antibody-3117
https://www.thermofisher.com/antibody/product/CD127-Antibody-clone-A7R34-Monoclonal/17-1271-82
https://www.bdbiosciences.com/en-ca/products/reagents/flow-cytometry-reagents/research-reagents/single-color-antibodies-ruo/buv737-rat-anti-mouse-cd127.612841
https://www.thermofisher.com/antibody/product/Ly-6G-Ly-6C-Antibody-clone-RB6-8C5-Monoclonal/12-5931-82
https://www.thermofisher.com/antibody/product/Ly-6G-Ly-6C-Antibody-clone-RB6-8C5-Monoclonal/A15424
https://www.thermofisher.com/antibody/product/KLRG1-Antibody-clone-2F1-Monoclonal/46-5893-82
https://www.thermofisher.com/antibody/product/CD335-NKp46-Antibody-clone-29A1-4-Monoclonal/25-3351-82

Animals and other organisms

Policy information about studies involving animals; ARRIVE guidelines recommended for reporting animal research

Laboratory animals

Balb/c Tbx21-/-Rag2-/- double KO (TRUC) mice have been described previously (Powell et al., 2015; Powell et al., 2012). Tbx21-/-Rag2-/-Il22-/- (TRUCIl22-/-) triple KO mice were generated by backcrossing Balb/c Tbx21-/-Rag2-/- double KO (TRUC) mice with Balb/c Il22-/- mice that were provided by Pfizer. C57Bl/6 WT and Rag1-/- mice were purchased from Charles River Laboratories. Il10-/- mice were provided by Professor Werner Muller, Faculty of Life Sciences, University of Manchester. Mice were housed in specific pathogen free (SPF) conditions. All mice were handled according to local (King's College London) and national guidelines, and all our experimental protocols were reviewed and approved by our local ethics review committee. All animal experiments were conducted in accredited facilities in accordance with the UK Animals (Scientific Procedures) Act 1986 (Home Office license number PPL 70/7869). All animals were housed at the KCL Biological Services Unit (BSU). A twelve hour light/dark cycle was implemented with the a slow rise and dim of lights over a half hour period. Temperatures range between 20 and 23 degrees Celsius and humidity 45% to 55%.

Wild animals

No wild animals were used for this study

Field-collected samples

No field-collected samples were used

Ethics oversight

King's College London, Home Office license number PPL 70/7869

Note that full information on the approval of the study protocol must also be provided in the manuscript.

Human research participants

Policy information about studies involving human research participants

Population characteristics

There were 550 patients with a diagnosis of ulcerative colitis recruited in the phase three registration clinical trial of ustekinumab in UC. The median age was 42 years with 35% being female and 65% male. The median Mayo score was 9, range (8,12) and the median disease duration was 6 years, range (3,12). Half of patients (50%) had previous exposure to anti-TNF therapy. The median CRP was 4.6mg/L, range (1.4, 12). The median faecal calprotectin (ug/g) was 1310, range (560, 2594).
Samples for colonoid development were collected from from six adult individuals (median age: 48, range[33,67], female:3), without past medical history or regular medication who attended for routine colonoscopy in view of abdominal symptoms without a diagnosis of IBD and did not have macroscopic or microscopic evidence of inflammation.

Recruitment

Previously reported (NEJM 2019)

Ethics oversight

Ethical approval for human samples used for colonoids were provided by King's College London, Guy's and St Thomas' NHS Foundation Trust and King's College Hospital. The national research ethics committee for England reviewed and approved the study protocol (IRAS id:190309). All patients provided samples after informed consent. No compensation was provided.
Ethical approval for the immunohistochemistry work on paraffin embedded tissue of patients was provided by the Newcastle Academic Health Partners Bioresource (Newcastle and North Tyneside 1 REC:12/NE/0395 & 10/H0906/41).

Note that full information on the approval of the study protocol must also be provided in the manuscript.

Clinical data

Policy information about clinical studies

All manuscripts should comply with the ICMJE guidelines for publication of clinical research and a completed CONSORT checklist must be included with all submissions.

Clinical trial registration	n/a
Study protocol	n/a
Data collection	n/a
Outcomes	n/a

Flow Cytometry

Plots

Confirm that:

- [x] The axis labels state the marker and fluorochrome used (e.g. CD4-FITC).
- [x] The axis scales are clearly visible. Include numbers along axes only for bottom left plot of group (a 'group' is an analysis of identical markers).
- [x] All plots are contour plots with outliers or pseudocolor plots.
- [x] A numerical value for number of cells or percentage (with statistics) is provided.

Methodology

Sample preparation	Mice were euthanized by either cervical dislocation or by a rising concentration of carbon dioxide gas, and then dissected in a laminar flow cabinet under aseptic conditions. Colons were opened longitudinally, cleaned thoroughly with ice-cold PBS and cut into 1-2mm pieces and washed with 10ml 5mM EDTA, 1mM Hepes in HBSS (Gibco) in a shaking water bath (300rpm) at 37°C for 20min. Tissue was then vortexed vigorously for 10sec and passed through a 100µM cell strainer and collected in C-tubes (Miltenyi) in complete RPMI (Gibco) containing 10% fetal calf serum, 0.25mg/ml Collagenase D (Roche), 1.5mg/ml Dispase II (Roche) and 0.01µg/ml DNase (Roche) and put in a shaking water bath (300rpm) at 37°C for 40min. Before and after the 40min incubation C-tubes were vigorously shaken for 30sec. Solutions were then passed through 100µM cell strainers and washed with ice-cold PBS. Cells were resuspended in 10ml of the 40% fraction of a 40:80 Percoll (GE Healthcare) gradient and carefully placed on top of 5ml of the 80% fraction in 15ml tubes. Percoll gradient separation was performed by 20min centrifugation at 2600rpm at room temperature without break. LP cells were collected from the interphase of the gradient and washed with ice-cold PBS. Cells were resuspended in 1ml PBS, counted and immediately used for further experiments.<br>Single cell suspensions were washed with ice cold PBS and centrifugation at 1800rpm, 4oC for 5min prior to all staining. Cells were then resuspended in 200µl PBS containing Fc block (aCD32/CD16, eBioscience) at 1:100 dilution and incubated on ice for 10min. Antibodies against all surface markers were added at appropriate dilutions as well as LIVE/DEAD Fixable Dead Cell Stain (Invitrogen) used in 1:1000. Samples were mixed by vortex and incubated for another 20min on ice in the dark. After the incubation, cells were washed with ice cold PBS and centrifugation at 1800rpm, 4oC for 5min and then fixed with 400µl of 4% PFA and incubated at RT for 15min in the dark. After fixation, cells were washed again with ice cold PSB, resuspended in 150-200µl PBS and stored at 4oC in the dark awaiting sample acquisition.<br>To obtain a pure population of naïve CD4+ T cells from the spleen, splenic single cell suspensions were first treated with ACK buffer for red blood cell lysis, enriched for CD4+ cells using immunomagnetic based cell separation and then stained with mAbs against CD4, CD25, CD44 and CD62L and LIVE/DEAD Fixable Dead Cell Stain (Invitrogen, UK) as described above. To obtain pure populations of colonic NCR- ILC3s, cLPMCs were stained with mAbs against CD45, CD90, CD127, KLRG1 and NKp46 and Live/Dead dye as described above.
Instrument	All samples were acquired on a BD LSRFortessaTM (BD Biosciences) at the Biomedical Research Council (BRC) Flow Core (15th Floor, Tower Wing, Guy's Hospital). All sorts were performed on BD Aria I, BD Aria II or BD Aria Fusion (BD Biosciences) at the BRC Flow Core (15th Floor, Tower Wing, Guy's Hospital).
Software	Data were analysed using FlowJo software (Treestar) and GraphPad Prism 8.
Cell population abundance	Purity checks were performed after every sort and purity was always found to be above 97%.
Gating strategy	Naïve CD4+ T cells were defined as live CD4+CD25-CD44loCD62Lhi cells. NCR- ILC3s were defined as live CD45+CD90+CD127+KLRG1-NKp46- cells.

- [x] Tick this box to confirm that a figure exemplifying the gating strategy is provided in the Supplementary Information.
